# Basics for sensorimotor information processing: some implications for learning

**DOI:** 10.3389/fpsyg.2015.00033

**Published:** 2015-02-16

**Authors:** Franck Vidal, Cédric Meckler, Thierry Hasbroucq

**Affiliations:** ^1^Laboratoire de Neurosciences Cognitives UMR 7291, Faculté des Sciences, Aix-Marseille UniversitéCNRS, Marseille, France; ^2^Institut de Recherche Biomédicale des Armées–Equipe Résidante de Recherche Subaquatique OpérationnelleToulon, France

**Keywords:** sensorimotor activities, reaction time, errors, learning, information processing

## Abstract

In sensorimotor activities, learning requires efficient information processing, whether in car driving, sport activities or human–machine interactions. Several factors may affect the efficiency of such processing: they may be extrinsic (i.e., task-related) or intrinsic (i.e., subjects-related). The effects of these factors are intimately related to the structure of human information processing. In the present article we will focus on some of them, which are poorly taken into account, even when minimizing errors or their consequences is an essential issue at stake. Among the extrinsic factors, we will discuss, first, the effects of the quantity and quality of information, secondly, the effects of instruction and thirdly motor program learning. Among the intrinsic factors, we will discuss first the influence of prior information, secondly how individual strategies affect performance and, thirdly, we will stress the fact that although the human brain is not structured to function errorless (which is not new) humans are able to detect their errors very quickly and (in most of the cases), fast enough to correct them before they result in an overt failure. Extrinsic and intrinsic factors are important to take into account for learning because (1) they strongly affect performance, either in terms of speed or accuracy, which facilitates or impairs learning, (2) the effect of certain extrinsic factors may be strongly modified by learning and (3) certain intrinsic factors might be exploited for learning strategies.

Whether in sport, car driving, music, or human–machine interactions, learning sensorimotor activities requires efficient information processing. Knowing some principles of human information processing may be useful to propose tasks and/or learning methods in conformity with these principles, so as to facilitate their realization and improve performance. Presenting some basics of these principles is the aim of the present article.

Even when the level of vigilance is optimal, the efficiency of such processing can be affected by extrinsic (task-related) or intrinsic (subject-related) factors. We call here “extrinsic” those factors on which the subject cannot act. Extrinsic factors may be related to the quantity of stimuli to be processed, the way decisions must be taken, the ability to exploit and consolidate appropriately motor programs or the availability of prior information regarding future events. We call here “intrinsic” those factors on which the subject can act. They may be related to strategic effects, voluntary orientation of attention (*in time and space*), motor preparation (*in time and space*) or action monitoring. In fact, intrinsic factors could be unified in terms of executive control processes (Norman and Shallice, 2000).

We will, first, discuss of extrinsic factors and, secondly, present intrinsic ones with a special emphasis on action monitoring processes.

## EXTRINSIC FACTORS

### STIMULUS-RELATED

We will not discuss here the effects of the quality of the stimuli since it is a commonplace that degraded or ambiguous stimuli impair perception and identification processes, which results in impaired performance.

#### Number of relevant stimuli

The number of stimuli to be processed is a critical determinant of performance.

If the stimuli are presented sequentially, increasing their number by time unit amounts increasing the time pressure put on the task, which will result in an increase of the error rate, according to the well established speed–accuracy trade-off (SAT; Fitts, 1966; Pew, 1969; Usher et al., 2002; Bogacz et al., 2009). We will briefly present SAT in section 2 (intrinsic factors).

In case several relevant stimuli must be presented simultaneously, increasing their number will force subjects to share their attention between these different stimuli, which will increase the reaction time (RT) and the likelihood to produce errors. This increase is explained by the fact that, all other things being equal, (1) processing of a relevant stimulus will interfere with processing of other relevant ones, because human information processing capacities are limited (Kahneman, 1973; Norman and Bobrow, 1975; Franconeri et al., 2013); (2) the mere fact to have one’s attention to be shared between several stimuli may often induces a cost, *per se* (Navon and Gopher, 1979). This cost may also be manifested by an increase in RT and/or an increase in error rate. Therefore, if performance is critical, attention sharing, when possible, should be avoided in sensorimotor activities (Hyman, 1953). We do not intend to discuss this point further since accurate and comprehensive reviews on this subject are already available (e.g., Wickens, 2002, 2008).

#### Number of irrelevant stimuli

When a single relevant stimulus (target) is presented among several irrelevant ones (odd stimuli), this stimulus must be selected among all the other ones. This selection process requires that attention is oriented toward the relevant stimulus while ignoring the odd ones.

In general terms, attention can be automatically captured (e.g., Posner and Cohen, 1984), or voluntarily oriented (e.g., Posner et al., 1980)^1^. The distinction between automatic and controlled processes has been theorized and empirically evaluated by the pioneering work of Schneider and Shiffrin (1977) and Shiffrin and Schneider (1977), and has revealed to be particularly useful in several research fields and especially in the field of attention. Several properties are assumed to oppose these two modes of processing: while automatic processes are supposed to be “fast, parallel, almost effortless … not limited by capacity … not under direct subject control” (Schneider et al., 1984, p. 1), controlled processes present the opposite properties. Regan (1981) has pointed a very important distinction between two meanings of the term “automatic”: involuntary, [i.e., unintentionally triggered and, when triggered, impossible to stop intentionally (Kahneman and Treisman, 1984)] and effortless [i.e., capacity-free and not subject to interferences (Kahneman and Treisman, 1984)]. Regan noted that these two properties may not necessarily be tied (Regan, 1981).

Regarding attentionnal processes, the important point lies in the “costless” nature of automaticity since, according to Schneider and Shiffrin (1977), it is admitted that a “special type of automatic process” can “direct attention automatically to a target stimulus” (Schneider and Chein, 2003, p. 527).

An example of this automatic capture can be found in the “pop out” phenomenon. Because elementary features of a stimulus are processed in parallel, if a target differs from odd ones by a single elementary feature, then, attention will be automatically captured at its location. As a consequence, the RT or the error rate will remain unaffected by the presentation of a target among odd stimuli, whatever their number (Treisman and Gelade, 1980; Nakayama and Silverman, 1986)^2^. In the visual modality, for example, these features can be color, orientation, brightness, size, depth, or direction of motion. Pop out effects can be accounted for by the fact that the visual structures process elementary features in (rather) separate specialized pathways (e.g., Livingstone and Hubel, 1988; Kastner et al., 1997; Treisman and Kanwisher, 1998, for a review).

Now, if the conjunction of two (or more) elementary features is needed to characterize a target among odd stimuli, the elementary features of the stimuli need to be bound to form unitary objects; attention would be responsible for this binding mechanism (Robertson, 2003), as metaphorically proposed by Treisman and Gelade (1980) “… focal attention provides the “glue” which integrates the initially separable features into unitary objects” (p. 98). In this case, voluntary attention must be directed serially to each stimulus of a display which results in a linear increase of RTs with the increasing number of odd stimuli, (Treisman and Gelade, 1980; Nakayama and Silverman, 1986). In this case, odd stimuli act as distracters.

This distinction between automatic and controlled modes of attention orienting is not only justified by the psychophysical properties of human performance; it is also supported by physiological data showing that the way structures involved in attention orienting (namely prefrontal and parietal areas) are recruited, differs in the automatic and controlled modes (e.g., Buschman and Miller, 2007).

From what precedes it appears that whenever possible, it is preferable to present information in such a way that attention is automatically oriented toward the relevant stimuli. Moreover, when this is not completely possible, the number of stimuli to be processed at a time should not be too high, especially when one has to learn new skills. Although this last point may seem trivial it is not always born in mind in man–machine interface designs or man–machine interactions. Operators are often presented with several stimuli corresponding to information that *might* be useful, even when these stimuli are useless most of the time (the implicit idea here seems to be that any possibly useful information must *always* be available and presented to the operator); as a consequence, if not necessary at a given moment, these stimuli will act as distracters, especially for non experts during task learning. This, for example, might be the case in certain head up displays.

Let us now consider reading: there is no time pressure *stricto sensu* in reading but most readers do it at the fastest pace compatible with a good comprehension of the text. Learning to read is a long and difficult process; it seems that for these reasons, textbooks for learning to read have taken into account the aforementioned principles, maybe intuitively or by trials and errors, but in any case, most often, each page of these textbooks is very accurately organized. First, pages present, (1) very large letters (allowing easy and fast processing), (2) a few number of relevant information by page (which avoids too much attention sharing) and (3) they present the to-be-learnt letters or association of letters in such a way that they pop out. Regarding this last point, for example, if the phoneme corresponding to a given letter (or to a group of letters such as “sh”) is the one to be learnt, then, the corresponding letter(s) is (are) generally written in bold or even in color, inside the word. As a consequence, the attention of the child is automatically (and effortlessly) driven to the relevant place(s) in the page and in the word.

These examples illustrate how simple principles regarding the organization of the stimuli to be processed may facilitate stimulus-related processing and, as a consequence, learning and performance.

### RESPONSE-RELATED

#### Position of the effectors and response programming

Most often, when responses are triggered under time pressure, they are executed in a ballistic mode. When these responses are well learnt, it is often assumed that they are executed *via* motor programs (Paillard, 1960). These programs can be viewed as sets of abstract instructions specifying explicitly certain characteristics of the responses to be produced (e.g., Keele, 1968; Requin et al., 1991). Several behavioral (Rosenbaum, 1980; Lépine et al., 1989) and physiological data in monkeys (Georgopoulos et al., 1986; Riehle and Requin, 1989; Riehle, 2005) or humans (MacKay and Bonnet, 1990; Leuthold and Jentzsch, 2009) suggest that these programs specify certain parameters of the incoming movement.

Except in certain specific cases where they are innate, motor programs emerge from practice. In other words, most motor programs are learnt and, as such, the issue of motor programs is relevant to motor expertise and learning.

A consequence of the concept of motor program is that once the execution of the program has begun, it goes on until its end. A consequence of the parametric conception is that, for the parameters to be efficiently set before response onset, the initial state of the moving effectors must be known accurately. It has been established for long that, for programmed responses, proprioceptive/tactile signals are poorly involved in the control of ongoing activities but that they are critical to build, consolidate or update motor programs, during practice (Keele, 1973). Moreover, proprioceptive and/or tactile information is critical for being informed (before the movement) on the initial position of a given effector (Ghez et al., 1990; Gordon et al., 1995) and it has been shown that inaccuracy of initial proprioceptive signals results in pointing errors (Vindras et al., 1998). This indicates that this type of information is critical to set the appropriate parameters of the motor programs.

The quality of proprioceptive/tactile information is not uniform across all limb positions (Rossetti et al., 1994). Fortunately, it is possible to easily identify optimal positions since those which are spontaneously judged as the most comfortable are also those which produce more accurate position signals (Rossetti et al., 1994). These comfortable positions are those which correspond to the median range of each joint position. This median range also corresponds to a minimization of medial muscle tension (Rossetti et al., 1994). It is likely that “… discomfort could … prevent the motor system from adopting postures where position signals are degraded, in order to keep the arm within a range of reliable encoding of posture” (Rossetti et al., 1994, p. 131).

As a consequence, a way to improve motor programs learning and/or motor programs execution is to improve the quality of initial proprioceptive and/or tactile information. Special attention must therefore be drawn on this point during the acquisition of motor skills. This is especially critical in young learners since proprioceptive information is less accurate in 6–8 children, as compared to 10–12 children: estimation of their static (initial) state is worse in the former than in the latter, which results in increased movement trajectory variability (King et al., 2012).

Although no clear explanation could be provided at that time, an example of application of these principles can be found in the famous method for keyboard playing by Bach (1753). Among the recommendations made by this musician, several concern the positioning of the arm, hand and fingers. Adopting these correct positions is critical for the future skills to be acquired as, according to Bach (1753), incorrect positions will never allow the learner to play correctly. The teachers should make the learner adopt comfortable or natural positions especially (1) with the forearm slightly lower than the keyboard (which corresponds to a median position for the wrist articulation), (2) with avoiding large movements of the arm (which would involve several articulation which errors of position are additive: Rossetti et al., 1994), which should move as little as possible (3) with adopting positions for which the fingers are flexed and not stretched; these flexed positions (which, as can be understood from Bach (1753) correspond to median positions of the finger articulations) should allow the muscles not to be stiff, stiffness being an obstacle to accurate, smooth and adequate playing (remember that medial positions have also the virtue to minimize medial muscle tension: Rossetti et al., 1994). Moreover, still according to Bach (1753), a negative side effect of fingers stretching is that the position of the thumb remains too far from the other fingers (in other words, the articulation of the thumb is in an extreme position, which is inappropriate for accurate position information). One can imagine that more systematic attention should be paid to these aspects for other motor skills acquisition such as sport skills.

Moreover, in man–machine interfaces, several sensorimotor tasks have to be performed by operators (for example, push as soon as possible a remote button in response to visual or auditory information – which is a prototypic pointing task). No care is usually taken regarding the relative position of the buttons as compared to the usual resting position of the hand. No much attention is paid on the possible (or recommended) resting/average positions for the arms and hands. Therefore, the operators cannot choose the most appropriate positions which are imposed by the task and the interface.

#### Sequential movements, motor programming and chunking

Motor programs are learnt and, for sequential movements (such as playing music), once they are initially learnt, even more learning can improve them.

This is (at least in part) assumed to be achieved through clustering together sets of elements of the sequence (e.g., Sakai et al., 2003). This clustering operation is also called “chunking.” Since activating motor programs takes time, chunking presents the advantage to pool together the elementary programs corresponding to each response of a sequence into a single program unit. Such an advantage is illustrated in the following example: in a simple RT task, although triggering a simple four-element finger movement sequence takes 62 ms longer than triggering a one-element simple finger response on a first day, this effect vanishes after 8 days training (Klapp, 1995). This effect of learning is explained in terms of chunking. When no choice is to be performed (simple RT task), subjects can choose and program the first element of a motor sequence before the onset of the response signal (RS; Klapp, 1974; Rosenbaum, 1980)^3^; as a consequence, the time necessary to program this first element does not contribute to RT. However, the other elements of the sequence must be programmed during the RT (and the time needed for this operation will increase the RT: Sternberg et al., 1978) and/or during the execution of the sequence, which may slower it. Therefore, chunking is a very efficient way to “compress” motor programs in order to save time when they are to be activated quickly. This cannot be done without training.

This interpretation has received support from physiological studies since, for example, the basal ganglia play a special role in chunking. Indeed, it has been shown that although it is possible to learn new sequences with basal ganglia impairment, chunking is no longer possible (Boyd et al., 2009; Tremblay et al., 2010).

Once again, it looks like keyboard players understood these principles, long before neuroscientists, since different learning methods seem to take chunking effects into account. Playing a keyboard is typically a sequential activity and, for example, Hanon (1873), in his famous “Le pianiste virtuose … (The virtuoso pianist …)” compilation of 60 exercises, recommends, when beginning to learn, to play one’s scale by stressing one (or two) note(s) every, say, 5–6 notes. This, of course produces a rhythm which is not actually in the score but, he says, this will facilitate learning. This facilitation can be interpreted in terms of chunking facilitation. Indeed, it is clear that these rhythms comprising 5–6 notes create artificial structures in the scale and allow grouping elements into chunks, which, as we mentioned earlier, is very efficient in term of fast sequence programming. We can see here that Hanon (1873) recommends (counter-intuitively) not to begin learning his exercises as they should be played; on the contrary, he takes into account first, what is better for learning and, only in a second step, what is desirable for playing.

Moreover, as indicated earlier, the activation of motor programs takes time. This is certainly why, Hanon or Bach recommend playing their exercises much slower than required on the score and, as learning goes by, to accelerate accordingly. This is a very efficient way to take into account that, with training (because of chunking), the number of to-be-programmed elements will decrease; as a consequence, the time needed to program (more exactly to activate the motor programs of) these elements will also decrease and will no longer be an obstacle to fast playing. Bach (1753) writes that by accelerating progressively, the finger placement will become so fluid that there is “*no need to think of it.”* In our terms one could translate (less artistically) the preceding words by: the finger movements of the different sequences of the piece are *“fully programmed.*”

### RESPONSE SELECTION

Even when stimuli can be identified effortlessly and when responses are very easy to execute, certain sensorimotor tasks may be very difficult to perform because certain stimulus-response associations (or, in other words, response selections), are very difficult to implement.

Response selection refers to determining which response alternative is required by the RS, and must be distinguished from response programming (more exactly motor program activation), which, as we just discussed in the preceding section, refers to organizing the selected movement. This determination process is, therefore, neither directly dependent on the stimuli, nor directly dependent on the responses and unit recordings in monkeys indeed evidenced neurons reacting neither to a response nor to a stimulus but selectively reacting to specific stimulus-response associations (Zhang et al., 1997). This process must rely on a stimulus-to-responses (S-R) mapping. This mapping will define the nature of the task and, of course, it is most often established by instruction. This last point is noteworthy since it is relevant to the issue of learning: a learner who is taught a task by his/her teacher will (in principle) comply with the instruction he/she is given.

#### Rule-based vs. list-based S-R mapping

There are (at least) two ways to establish a mapping relationship between a stimulus set and a response set: (1) a rule-based S-R mapping and (2) a list-based S-R mapping.

In case (1), a rule establishes the nature of the S-R mapping; for example instruction requires producing a right response to the presentation of even digits and a left response to odd digits. In case (2), instruction consists in an arbitrary list of digits-to-finger mapping; for example 0, 3, 4, 6, or 7 require a right response and 1,2,5,8, or 9 a left response. In case (1), the difficulty of the response selection process will depend on how easily this rule will be applied. In case (2) the difficulty of the response selection process will depend on the length of the list (Hasbroucq et al., 1990; Hasbroucq, unpublished). All other things being equal, it is easier to apply and learn a rule-based mapping than a list-based one (Hasbroucq et al., 1990; Hasbroucq, unpublished).

Response uncertainty has a very strong impact on performance. This can be experimentally manipulated by varying the number of response choices; it is well established that the RT and the error rate increase with the number of possible responses (Hick, 1952). Of course, the number of choices can affect each level of information processing: the perceptual level if, for example, uncertainty involves the different positions of the possible RSs, as well as the response level since only one among several possible response programs must be activated.

Regarding the response selection level, the effect of the number of alternatives depends on the nature of the S-R mapping: rule-based or list-based. It is clear that when list-based, the difficulty of the selection process will mainly depend on the size of the list from which the subject must retrieve, at each trial, the response corresponding to the presented stimulus. On the contrary, the process of applying a rule, being only dependent on the nature of the rule, rule application is not supposed to depend on the number of choices (Hasbroucq et al., 1990) and empirical evidence support this view (Hasbroucq, unpublished). Therefore, teachers, task prescribers or man–machine interfaces designers should be aware of these principles and try to apply them whenever possible.

#### Stimulus-response compatibility effects

Stimulus-response compatibility (SRC) effects can be defined as “… the modification of performance induced by a change in the SR [mapping] relationship that is uncorrelated with any change in stimulation or responding” (Hasbroucq and Guiard, 1991, p. 246). This definition is not theoretic but empiric and does not refer to any specific mechanism. However, since this type of effects cannot be accounted for by purely stimulus-related or purely response-related phenomena, it must be assumed that it affects response selection processes (e.g., Sanders, 1990). From this definition, it also appears that SRC effects only have to do with rule-based mapping. Let us consider a very simple case of between-hand choice RT task. Suppose that the stimuli consist in a visual dot presented on the right or on the left side of the subject. If subjects are instructed to respond on the same side as the stimulus (right and left responses to right and left stimuli respectively), the RT is shorter (Shaffer, 1966) than if subjects are instructed to respond on the side opposite to the stimulus (right and left responses to left and right stimuli, respectively). This simple case of spatial SRC effect indicates that, all other things being equal, certain associations between stimuli and responses are easier to apply. These concern instruction-based relationships.

#### Stimulus-response congruency effects

Now, it seems that irrelevant S-R transformations can also proceed automatically and influence RT, because overlearned. An example of these automatic, irrelevant, overlearned S-R transformations may be found in the Simon task (Simon, 1990; Lu and Proctor, 1995, for reviews). In a Simon task, subjects have to respond spatially (e.g., right or left; up or down …) to a non spatial feature of a RS (e.g., color, pitch …), while ignoring its spatial irrelevant feature. For example, subjects have to give a right response to a blue stimulus or a left response to a yellow one, regardless of the stimulus position (right or left). When the position of the required response and that of the stimulus correspond (congruent condition) the RT and the error rate are smaller than when response and stimulus positions are opposite (incongruent condition).

Although it is not warranted that the Simon effect involves response selection processes only (Hasbroucq and Guiard, 1991), response selection may be influenced by congruity (Carbonnell et al., 2013).

It seems that, in a Simon task, two S-R transformations are activated in parallel: a controlled instruction-based transformation plus an automatic one (always congruent, established by previous experience or, in other word, learning)^4^.

On congruent conditions, both the automatic and controlled S-R transformations activate the same (correct) response. On the contrary, on incongruent conditions, while the controlled S-R transformation activates the correct response, the automatic transformation activates the incorrect one. As a consequence, on incongruent conditions, these two S-R transformations will compete and the resolution of this competition (Kornblum et al., 1990) will take time (which increases the RT) and may sometimes fail (which results in increased error rate). In more complex situations, *a priori* naive judgements are not sufficient to determine which associations will be congruent and which ones will not (Payne, 1995; Vu and Proctor, 2003; Hoffmann, 2010).

Now, what learning has done (establishing automatic associations), learning can also undo it. If subjects are trained to perform a spatially incompatible RT task (i.e., respond right or left to left or right stimuli, respectively), and have to perform a Simon task after, the Simon effect vanishes (Tagliabue et al., 2000, 2002).

Before practice, tasks are often explained to learners by instruction; instruction being usually the first event when teaching a task. Instructions alone can establish or abolish stimulus-response association without practice. If subjects must pronounce the meaningless syllables “bee” or “boo” in response to the presentation of words meaning either right or left and, in other trials, must give a “bee” or a “boo” response to blue or green squares, respectively, RT is faster when the blue square is presented to the right than when it is presented to the left: an instruction-based Simon effects appears (De Houwer et al., 2005). Conversely, if a preparatory instruction indicates which mapping (compatible or incompatible) should be applied for a spatial task following a Simon task, in the same trial, the regular Simon effect vanishes on the (first) Simon task, if the instructed mapping of the spatial task is incompatible (but persists if the spatial task mapping is compatible; Theeuwes et al., 2014).

To sum up: (1) certain S-R mapping rules, equally easy to understand (e.g., respond on the same side as the stimulus, or respond on the side opposite to the stimulus), are not equally easy to implement; (2) given a S-R mapping rule (e.g., respond left to blue stimuli or right to yellow ones), certain S-R associations may facilitate (congruent associations) or impair (incongruent associations) response selection processes and, as a consequence, performance; (3) the congruent, incongruent (or neutral) nature of a new S-R association cannot be determined naively but must be established empirically, (4) the way learners are instructed to perform a task has a critical influence on response selection processes (e.g., Tandonnet et al., 2014) since, (a) instructions establish S-R associations, (b) instructions alone can induce congruency effects, (c) instructions alone can abolish congruency effects.

### INTRINSIC FACTORS

Dependent on the structure of tasks, advance information regarding the to-be-performed responses is available or not. This prior information may allow subjects to orient appropriately their attention, to select in advance the appropriate response, to program completely or partially their response. Of course, this depends on the structure of the task and, as such, availability of advance information belongs to extrinsic factors. Now, subjects can decide to use or not to use advance information and, as such, the effects of advance information also depend on intrinsic factors. Because, the effects of advance information as well as “purely” intrinsic factors can be unified in terms of executive control, we chose to discuss them in this section.

### PREPARATION

Advance information is supposed to reduce subjects’ uncertainty regarding forthcoming events. This information can be relative to events proper (for example, the nature of a RS). This information can also be relative to timing (for example the moment of occurrence of a RS). Therefore, when prior information is available, it is possible to get prepared, either to *which* event(s) will occur or to *when* event(s) will occur, or both. This preparation can be evidenced by the fact that RT is much faster when preparation is possible than when it is not. Depending on the nature of advance information (regarding “what” or regarding “when”), Requin et al. (1991) distinguished two, not mutually exclusive, types of preparation: event preparation and time preparation.

#### Event preparation

If a soccer goal-keeper facing a penalty shoot moves toward one side before the shooter kicks the ball, this information will be used by the shooter to score on the other side. Now, if the goal-keeper waits for the kick and, then, tries to react appropriately, he/she will never stop the ball because his/her RT will be too long. Therefore, goal-keepers make a bet, “choosing” one side in advance and, if the shooter happens to shoot at this same side, they get a chance to stop the ball. What do journalists actually mean when saying that a goal-keeper who stopped a penalty shoot has “chosen” the right side? They mean that (according to which his coach taught him), he/she selected one side and prepared a jump toward this side. As a consequence, his/her RT has been short enough to allow him/her having his/her hand at the right place on time, and stop the ball^5^. This example illustrates the benefits of event preparation in the domain of response preparation.

***Responses.*** But what do we mean when we say that the goal-keeper has “prepared” a jump in a predetermined direction? An important observation is that event preparation reduces RT. Therefore, either processing speed regarding some operations increases when preparing or, at constant processing speed, certain operations are done in advance and are not required anymore after the RS, which reduces RT.

First, the goal keeper can, at least, decide in advance toward which side he/she will react. In other words, the selection process can be achieved before the ball is kicked and this contributes reducing RT. But what about motor programs?

When a response is programmed, it has necessarily been selected. Therefore, selection and programming operations may often be confounded factors. To disentangle selection and motor programming, a solution proposed by Klapp et al. (1974) is to evaluate motor programming while maintaining constant the number of choices. Klapp et al. (1974) wondered whether the duration of a motor response could be a specified parameter of motor programs. In a RT paradigm, subjects had to produce either a short (150 ms) or a long (600 ms) keypress. In choice RT conditions (where no program can be prepared), RT was shorter before a short than a long keypress. On the contrary, if a cue indicated before the RS which response (short or long) should be produced, no RT difference was found between short and long responses. The authors therefore concluded that the short–long differences in choice RT revealed differences in the times needed to program a short and a long response. The absence of such an effect when the same responses were precued indicated that, in this case, programming operations had taken place before the RS, and, consequently, did not influence RT anymore. This interaction between response duration and conditions (uncuded *vs* precued response duration) was a strong argument in favor of the idea that, with prior information, motor programming can occur in advance. In other words, what has been done during the preparatory period (PP; programming) has no longer to be done after the RS (which contributes reducing RT). Note that, in Klapp et al.’s (1974) paradigm, there was no confounding factor (e.g., selection) with response programming. The short-long effect has been reproduced several times by different teams (e.g., Zelaznik and Hahn, 1985; Vidal et al., 1995).

Klapp et al. (1974) inferred from the RT pattern they observed that response duration was prepared before the RS when precued. Vidal et al. (1995) provided physiological support in favor of this view. They recorded electroencephalographic (EEG) activity over motor areas in humans during the PP of a precueing (Rosenbaum, 1980) paradigm where subjects had to produce either a short (700 ms) or a long (2500 ms) interval delimited by two brief button presses. Two seconds before the RS, a precue could either indicate which response (short or long) should be produced, or give no information regarding response duration. The short–long effect was reproduced. Moreover, during the PP, EEG activity was small when the cue gave no advance information, and larger when the cue indicated which response to produce. However, this simple effect could be attributed to motor programming, response selection or both. The important finding was that, when the cue indicated which response to produce, EEG activity was larger for the short than for the long response during the PP. In other words, when duration programming was assumed to occur during the PP, a short–long effect was observed on the amplitude of preparatory EEG activity. It is to be noted that this effect was observable over the supplementary motor areas (but not over the primary motor areas) in the early part of the PP, while the same effect showed up in the very late part of the PP over the primary motor areas (but not over the supplementary motor areas anymore). This latter point suggested that duration programming had taken place (at least in part) in the supplementary motor areas with advance information and that the program had been later transferred to the primary motor areas, just before the “go” signal, for execution.

Unit activities recordings from monkeys also support the view that response parameters can be programmed in advance. For example, Riehle (1991) could record primary motor cortex neurons which activity was phasically related to the precue when this signal indicated in advance which response was to be executed and these neurons did not react after the RS. On the contrary, when the precue gave no advance information regarding the response, these neurons were almost inactive after the precue but phasically discharged after the RS (which, in this case, gave all information regarding the response to be produced). Once again, the activity of these neurons suggests that what had been done before (shortly after the precue), has no longer to be done after the RS; with uninformative precues, this still needed to be done just after the RS.

Coming back to the goal keeper, it seems quite safe to conclude that, when ≪ choosing ≫ a side, he/she selects this side first and programs all the initial sequence of his/her jump. These two processes do not contribute to his RT anymore; as a consequence, his RT is shorter, which increases his/her chances to stop the ball.

***Stimuli.*** Preparatory information regarding events may also improve operations upstream programming and selection processes, that is, stimulus processing. This has been clearly and elegantly demonstrated by Posner et al. (1980). In a RT task, subjects had to respond as soon as possible by a keypress after a RS which could be presented to the right or to the left of a fixation point. Before the RS, an arrow pointing to the right or the left indicated on which side the RS was more likely to occur: in 80% of the trials, the RS occurred on the side indicated by the arrow (valid trials) and in 20% of the trials, the RS occurred on the other side (invalid trials). Although subjects had to keep their gaze oriented on the fixation point, RT was shorter in valid than in invalid conditions. The important point to be noticed, here, is that the response to be produced was always the same. Therefore, the decrease in RT could not be attributed to advance selection or programming processes. Posner et al. (1980), interpreted their results in term of attentional effects: covert attention was oriented on the side indicated by the arrows. This allowed faster detection of the stimuli in the valid condition, at the cost of slower detection (as compared to a neutral condition) in the invalid condition. These effects have been reproduced several times ever since (e.g., Coull and Nobre, 1998). They demonstrate how voluntary orientation of attention improves stimulus processing, provided that attention is appropriately oriented.

#### Time preparation

At Atlanta’s 1996 Olympic Games, British sprinter Linford Christie false-started twice in the men’s 100 m final and was disqualified from the competition. He refused the decision, remained on the track and delayed the final for several minutes. At Paris’s 2003 world championship, American sprinter Jon Drummond also false-started in a 100 m quarterfinal. Drummond also contested the decision and stayed on the track repeatedly shouting “I did not move.”

What happened to these champions illustrates an interesting aspect of time preparation. Before a 100 m race, there is no uncertainty regarding the “what,” which can be fully prepared. Preparing the “when” would also reduce RT but, since the delay between the “get set” order and the “go” signal is *a priori* unpredictable, it seems that time preparation is impossible in this situation. However, since this delay cannot last very long, as time goes by after the “get set” order, the probability that the “go” signal will occur “right now” increases progressively and the level of preparation may increase accordingly. One can therefore imagine that these two sprinters, under strong motivation, prepared as well as possible the motor sequence corresponding to their start but also got prepared for the moment when the “go” signal would occur, so much prepared that refraining moving began impossible too early; they probably did not feel that they had anticipated the go signal (which, it seems, they actually did).

The case of the abovementioned sprinters is particular because they prepared their move and (to a certain extent) the moment to move. Although counter intuitive, it is also possible to prepare the moment without being able to prepare the response. For example, in choice RT tasks in which the moment of occurrence of the RS is predictable, RT is faster than when the occurrence of the RS is not (or less) predictable (Requin et al., 1991, for a review). However, the locus of the effect of time preparation is still a matter of debate. In the following, we will provide arguments indicating that time preparation affects (at least) sensory and motor processes^6^.

***Responses.*** Absolute accuracy of time estimation decreases as the duration to be estimated increases (Gibbon, 1977). As a consequence, preparation is better timed for short foreperiods than for longer ones. This would explain why RT increases with increasing foreperiod duration^7^, provided that each foreperiod duration is administered in different blocks of trials (Woodrow, 1914). Therefore, a convenient way to manipulate time preparation is to manipulate the duration of PPs across blocks of trials.

It has been shown that time preparation modifies the excitability of corticospinal pathways during the PP (e.g., Davranche et al., 2007). However, these preparatory effects do not demonstrate that motor processes are speeded up. Therefore, Tandonnet et al. (2003, 2012) examined the time course of activation of the primary motor cortex during the RT period following short or long foreperiods, with EEG and transcranial magnetic stimulation (TMS). In a between-hand choice RT task, Tandonnet et al. (2003), used Laplacian-transformed data which allowed them to examine EEG activities of the primary motor cortex contralateral to the responding hand (M1). The authors showed that the time separating M1 activation from EMG onset was shorter after a short (500 ms) than a long (2500 ms) foreperiod. This indicated that better time preparation (during short foreperiods) speed up motor processes. In other words the authors showed that there is (at least) a motor locus for the effects of time preparation. In the same vein, Tandonnet et al. (2012), using TMS, examined the time course of excitability of the primary motor cortex contralateral to the responding hand in a between-hand choice RT task following the same foreperiods. The authors showed that, after the RS, the amplitude of the motor evoked potential of the responding hand increased earlier following the short than the long foreperiod. Note that such an effect was absent for the non-responding hand. Moreover, the dissociation between responding and non responding hands occurred earlier after the short than after the long foreperiod. This again indicated that the required response is implemented faster in the corticospinal system when time preparation is more efficient. As a consequence, it seems that time preparation speeds up motor processes (even when event preparation is impossible), which contributes improving performance.

***Stimuli.*** Time preparation can also improve stimulus processing. Coull and Nobre (1998) examined the effect of time preparation on stimulus processing using a temporal variant of the Posner et al.’s (1980) task. In a simple RT task, subjects had to respond as soon as possible by a keypress after a RS which could be presented to the right or to the left of a fixation point. Before the RS, a preparatory signal could prime the duration (short: 300 ms or long: 1500 ms) of the foreperiod (i.e., the moment when a RS would be presented). This prime was valid in 80% and invalid in 20% of the trials. RTs were faster for validly than invalidly primed foreperiods. However, this effect was much larger for invalid long primes and modest (or even absent) for invalid short primes. The authors convincingly argued that when subjects have anticipated a long foreperiod, the RS occurs too early and finds subjects in an unprepared state, which impairs their performance. On the contrary, when subjects have anticipated a short period and the stimulus has not occurred after a time corresponding to the end of the short foreperiod, subjects necessarily know that the stimulus will occur later, at the end of the long foreperiod. In this case, they can reorient their attention during the waiting delay, which allows them maintaining their performance.

Note that, in this experiment again, only one response was possible; therefore RT variations could not be attributed to selection and/or programming operations. Subject only had to detect the signal and, then, execute the pre-selected and pre-programmed response. Now, keeping in mind that time preparation can speed up motor processes (Tandonnet et al., 2003, 2012), it could well be that the RT advantage provided by time preparation was in part or completely due to motor improvement (speeding up). However, Davranche et al. (2011), in a detection task without any time pressure, demonstrated that time preparation actually facilitates stimulus detection for precued short foreperiods.

Coming back, now, to false start 100 m races, it is clear that, although accurate event preparation is encouraged, it seems that time preparation is prohibited. Indeed, Linford Christie was disqualified not because he moved before the go signal but because he reacted too fast (60 ms). RTs inferior to 100 ms are considered as false starts because it is assumed that reacting in less than 100 ms is not physiologically possible. Let us indicate here that we feel that this opinion is not firmly physiologically grounded and appears somehow arbitrary. If one really wants to discourage time anticipation, there are psycho-physiologically efficient ways to avoid it. Instead of having the go signal given by a human operator, it could easily be given by an automatic mechanism using so called “non aging” probability functions to determine the occurrence of go signals. Such a procedure would render almost impossible time preparation and, therefore, would probably avoid these recurrent false start problems.

To sum up, as soon as any type of information is available, whether it concerns the nature of the incoming events or the moment when something will occur, the human brain adapts in advance. By doing so, it anticipates future events thereby avoiding that adaptation takes place under time pressure, in reaction to these events. In other words, preparation processes can help release time pressure since all computations done in advance don’t need to be repeated during the time left to react. Therefore, when relevant events occur, the “reactive” processing cost is lower thereby increasing processing efficacy. Several examples of anticipatory adaptation to predictable events can be found in other domains of physiology such as blood pressure regulation, thermoregulation or regulation of glycemia.

Event and time preparation are of prominent importance for several activities that must be learnt such as, for example,driving, sport, hunting, or music.

In driving, a large part of teaching consists in making learn what must be anticipated. On the event side of preparation one can cite (non exhaustively): learning to get prepared to the nature of the road given the corresponding road signs, get prepared to the reactions of the car to the drivers’ actions, get prepared to the possible moves of the other drivers … On the time side of preparation one can cite (non exhaustively): learning to get prepared to the time it takes to stop after breaking, get prepared to the moment when the traffic light will turn red/green, get prepared to the deceleration in a very close future of the preceding vehicle when the braking lights turn on ….

In a similar way a hunter, before becoming a good hunter, must learn to read (interpret) the signs available in nature which allow him/her to anticipate and get prepared to what the game might do and when it might to it.

In team sports, a large part of learning also consists in becoming able to “read” the game. Reading the game amounts to being able to anticipate the moves of the other players, get prepared and act quickly in consequence. This preparation relates, not only on *what* the other players will do (or will not do) but also relates on *when* they might do it. In certain sports, such as rugby football, certain game sequences are completely repeated and learnt before the matches. This training will not make the players, say, run faster. However, it will allow accurate execution of motor programs (see preceding section) and, overall, full preparation of each player to the different events of the sequence. This full preparation gives them a significant advantage as compared to the adverse players who cannot do better than react to this sequence – except if the adverse coach has studied videos of previous matches and trained his own team to anticipate for such sequences (if they are used too often). Anticipation is also critical in other sports such as combat sports or any sport in which, reading and anticipating opponent’s actions provides an serious advantage (e.g., tennis).

Finally, in music, temporal preparation is critical: anticipation of rhythmic patterns is essential for musical expectancy and, of course, for accurate performance. Moreover playing instruments presenting a certain “inertia” (for example there is a non negligible delay between the moments when a musician blows in a tuba and the moment when the corresponding sound is produced) needs that the musician learns (and is prepared to) this inertia before being able to play in an orchestra. Event preparation is also important and must be learnt in music. When playing together, musicians performing music with a sketchy outline voluntarily exchange non-verbal cues; these cues act as preparatory signals and can be non temporal (specific movements, face expressions, glances …) or temporal information. For example, jazzmen, in their communication, learn to exchange temporal cues which take the form of subtle deviations from the well-established rhythmic pattern (Vuust et al., 2005).

### STRATEGIC EFFECTS: SPEED–ACCURACY TRADE-OFFS

Among strategic effects SATs are well documented. As we will see below, it is possible to trade speed against accuracy and accuracy against speed. Long term (between experimental conditions) strategic changes are called macro trade-offs, while short term (from trial to trial) changes are called micro trade-offs (e.g., Jentzsch and Leuthold, 2006).

#### Macro trade-offs

As indicated in the first section, increasing time pressure by constraining the time let for giving the appropriate response after a RS, results in an increase in error rate. This is an extrinsic constraint. Now, subjects can also choose to increase speed or accuracy spontaneously or, for example, to comply with experimenter or teacher instructions. If they increase speed, this will be at the cost of increasing the error rate. Conversely if they choose to increase accuracy, this will be done at the cost of increasing RT. In other words, subjects can trade accuracy against speed and conversely. This trade-off takes the form of an *S*-shaped curve. This phenomenon has been called SAT (Fitts, 1966; Pew, 1969). This phenomenon can be used for training and, according to the performance pattern displayed by learners, teachers can encourage their trainees to favor speed or accuracy at different stages of learning and performance. Indeed, subjects comply quite easily with speed/accuracy instructions.

#### Micro trade-offs

In choice RT time tasks, as mentioned above, errors may occur. RT of errors is often (but not always) faster than that of correct responses (Smith and Brewer, 1995). After errors, the RT is longer (post-error slowing) than after correct responses (Rabbitt, 1966; Smith and Brewer, 1995; Allain et al., 2009) and the error rate may also be lower (Laming, 1979; Ruitenberg et al., 2014). On the contrary, before errors, RT is shorter (pre-error speeding) than before a correct response (Smith and Brewer, 1995; Allain et al., 2009; Dutilh et al., 2012a). This saw tooth pattern of RT suggests that these sequential adjustments are strategic and reflect trial-by-trial micro trade-offs (Smith and Brewer, 1995). After an error, subjects would adopt a more cautious strategy (Laming, 1979; Dutilh et al., 2012b; Ruitenberg et al., 2014; but see Notebaert et al., 2009 for alternative accounts) and on the subsequent trials, subjects becoming more and more confident would progressively speed up until an error is generated again. These micro trade-offs suggest that an action monitoring system is at work at each trial and, as a function of performance, regulates responding behavior in order to optimize it (Botvinick et al., 2001). Therefore, these micro trade-offs would reveal trial by trial, “off line,” action monitoring.

The implication of post-error slowing in learning is not obvious; however, it is possible that this type of adjustment reflects a fast short-termed learning process from the preceding error.

### ON LINE ACTION MONITORING

In the preceding section, micro trade-offs suggest that an action monitoring system triggers behavioral adjustments to optimize performance. These adjustments occurring in reaction to errors, we can qualify them as “reactive.” Of course, these reactive adjustments occur *between* trials. One could wonder whether such reactions to errors might also occur *within* trial.

#### Reactive action monitoring

***EMG indices.*** In a between-hand choice RT task, Allain et al. (2004) recorded surface electromyographic (EMG) activity of the prime mover muscles involved in the two possible responses. They showed that the amplitude of the erroneous EMG bursts was smaller than that of the correct ones The initial slope of these EMG bursts being identical on correct responses and on errors, it is likely that the initial motor command was identical in both types of responses. Therefore, it seems that the smaller EMG bursts observed on errors reflect an inhibition of the motor command during its execution. If this interpretation is valid we can conclude that once the error is triggered, the action monitoring system detects it and reacts in an attempt to stop it. Now, considering that these errors were committed, it seems that the action monitoring system failed. What could, therefore, be the functional significance of this EMG effect?

Coles et al. (1985) showed that, in between-hand choice RTs tasks, certain correct responses are preceded by a small sub-threshold EMG activation (insufficient to trigger an overt response) on the “wrong” side. The RT of these correct trials is longer than that of correct trials which do not contain sub-threshold incorrect EMG activations (termed “pure” correct trials, in the following). These observations have been reproduced several times ever since (e.g., Allain et al., 2009).

There is a general agreement that, since these sub-threshold activations occurred on the “wrong” side, they correspond to partial errors. Burle et al. (2002b) proposed, in addition, that these partial errors correspond to almost errors that have been detected, inhibited and corrected on time. Allain et al. (2009) provided arguments in favor of this view, showing that sequential speeding and slowing adjustments also exist before and after partial errors respectively, although the size of these adjustments is smaller for partial than for full blown errors. Moreover, the fact that RT is longer on partial error trials than on pure correct trials could be a sign that, after a partial error, a part of processing operations must be done again in order to produce the correct response.

If these interpretations are correct, the existence of partial errors indicates that the action monitoring system is most often able to detect, inhibit and correct erroneous motor commands. When it works, this results in a partial error; when it fails this results in a full blown error; however, on errors, a sign of the tentative inhibition implemented by the action monitoring system can be found in the smaller size of the EMG bursts as compared to pure correct responses. Finally, still if these interpretations are correct, it is possible to calculate an index of the efficiency of the action monitoring system: the correction ratio, which corresponds to the ratio between successfully corrected incorrect activations (partial errors) and the total amount of incorrect activations (partial errors plus full errors; Burle et al., 2002b). For example, in the Simon task, the error rate is higher on incongruent than on congruent trials, as indicated above. However, a part of this effect is due to a decrease of the correction ratio on incongruent trials. Therefore, a part of the increase of the error rate on incongruent trials is due to a lowered efficiency of the action monitoring system. This indicates that there are two (not mutually exclusive) ways to deteriorate performance in terms of accuracy: decreasing the efficiency of the information processing chain, or decreasing the efficiency of the action monitoring system (or both, as it is the case on incongruent trials of a Simon task).

***EEG indices.*** Falkenstein et al. (1991) discovered that a large response-locked event-related potential (ERP) was evoked by errors. Since this ERP seemed specific to errors, it has been called error negativity (Ne) or, later on, error-related negativity (ERN: Gehring et al., 1993)^8^. Scheffers et al. (1996) showed that an Ne was also evoked by partial errors, although its amplitude was smaller than on errors. This observation makes sense if one admits that partial errors are almost errors. Finally, using Laplacian-transformed data, Vidal et al. (2000) unmasked a small Ne-like on correct trials, too, that had been unnoticed so far. It is clear that the discovery of the Ne constituted strong evidence for the existence of an action monitoring system able to quickly^9^ separate errors from correct responses at the very moment of the response. However, revealing the existence of an Ne-like on correct responses, also raised the question of the relationship between the Ne and the Ne-like waves: if the Ne-like is just a small Ne, then current models of action monitoring need to be revised to incorporate this finding that they cannot account for; if the Ne-like corresponds to another component, the Ne is specific to errors (full blown or partial) and there is no need for such a revision. Recent data (Bonini et al., 2014) suggest that the Ne and the Ne-like share (at least) a common source: the supplementary motor areas proper (SMAp). This finding suggests that the Ne-like and the Ne correspond to a same component which is modulated in amplitude: small on correct responses, larger on partial errors and even larger on full blown errors. Although this sensitivity of SMAp to performance is a sign of the existence of an action monitoring system, as initially shown by Falkenstein et al. (1991), its functional significance is not obvious. It has been proposed that SMAp could generate warning “default” signals which amplitude would depend on the ongoing performance (Bonini et al., 2014).

The sensitivity of SMAp to performance, just after EMG onsets, corresponds to another piece of evidence that the action monitoring system reacts *within* trial to the quality of performance.

#### Proactive action monitoring

In the preceding sections, we have seen that EMG and EEG data indicate that the action monitoring system *reacts* to incorrect activations in an attempt to correct them and avoid full blown errors. One could, finally, wonder whether the action monitoring system could *act before* incorrect activations occur in an attempt to prevent them.

Hasbroucq et al. (2000), using H reflex showed that, in a between-hand choice RT task, the excitability of spinal motoneurons corresponding to the responding hand increased, while it decreased on the other side, just before response execution. Burle et al. (2002a), using TMS showed that, in a between-hand choice RT task, the excitability of the primary motor cortex contralateral to the responding hand (contra M1) increased before response execution, while that of the primary motor cortex ipsilateral to the responding hand (ipsi M1) decreased. Finally, using Laplacian-transformed EEG recordings over contra and ipsi M1, Vidal et al. (2003) reported, in a between-hand choice RT task, a response-locked negative/positive pattern, likely corresponding to an EEG counterpart of the activation/inhibition pattern previously evidenced with other techniques by Hasbroucq et al. (2000) and Burle et al. (2002a). While it had previously been shown in monkeys that the negativity observed over contra M1 corresponds to the activation of the apical dendrites of layer V of contra M1 (Arezzo and Vaughan, 1980), the nature of ipsilateral inhibition appeared less clearly. Considering that, before spontaneous movements, contra M1 activation is present but not ispi M1 inhibition (e.g., Ikeda et al., 1995), Vidal et al. (2003) proposed that this inhibition was needed for preventing the emission of the “wrong” response. In other words, ipsilateral inhibition could represent the existence of an *a priori* mechanism of error prevention set proactively by the action monitoring system. In a go no-go task, errors are possible but, contrary to choice RT ones, not with the non responding hand. Vidal et al. (2011) showed that ipsilateral inhibition was absent in a go no-go task, which was in line with the interpretation of ipsi M1 inhibition by Vidal et al. (2003). Finally, in a between-hand choice RT task, Meckler et al. (2010) manipulated the risk of committing errors. They compared a “classical” choice RT with a biased one. In the classical condition, each response was equiprobable (50%) while in the biased one, one of the two responses was 80% probable (and the other one 20%, only). In the 80% condition, the response was expected, in the 50% condition there was no specific expectation, while in the 20% condition the response was unexpected. RT and error rates increased from expected to no expectation conditions and from no expectation to unexpected conditions.

Contra M1 activation was not affected by expectation. On the contrary, ipsilateral inhibition was small in the expected condition, larger in the no expectation condition and even larger in the unexpected condition. Meckler et al. (2010) concluded that ipsilateral inhibition was actually set to prevent errors, especially when considering that, in the unexpected condition, there was an inverse (between-subjects) correlation between the strength of ipsilateral inhibition and the error rate. In other words, in the condition where the risk of committing an error was highest, those subjects whose inhibition was stronger were those who committed the smallest proportion of errors.

Therefore, it seems that ipsi M1 inhibition is a physiological marker of a “double-checks” proactive action monitoring mechanism. Whether or not this proactive mechanism develops during the task by training is not known, yet. However, the possibility to develop this inhibition needs a certain degree of maturation. Van de Laar et al. (2012) showed that ipsi M1 inhibition was neither present in 8-years old nor in 12-years old children. It is worth noticing that the absence of inhibition in children was associated to almost 3 times (2.9) more partial errors and 3 times more errors than in 20-years old young adults. Although Van de Laar et al. (2012) did not address or discuss the following point, it is easy from their Table 2 (which reports partial and full error rates) to calculate correction ratios (Burle et al., 2002b). A striking result appears: young children corrected their incorrect activations in 75% of the cases while young adults corrected them in 76% of the cases. Although no statistical data are available in Van de Laar et al. (2012), it is very likely that correction rates of children and adults did not differ. As a consequence, it is not action monitoring as a whole which is deficient in children: their high error rate cannot be attributed to reactive action monitoring failure, as revealed by partial errors. It seems, on the contrary, that their deficiency must be found, at least in part, in immature proactive action monitoring, as revealed by their lack of ipsilateral M1 inhibition.

To sum up, recent research showed that, even under time pressure, sensorimotor information processing may not be completely unidirectional: reprocessing can quickly be triggered by the action monitoring system in reaction to incorrectly activated responses. These reactive mechanisms allow avoiding several overt errors. Moreover, not only does the brain react to incorrect activations but it also anticipates them and sets preventive mechanisms to avoid them in error-prone situations. It is noteworthy that that the reactive side of the action monitoring system is efficient quite early in childhood, but that its proactive side needs a longer maturation to optimize performance in sensorimotor activities.

From what precedes it is quite clear that the human brain is not structured to function errorless, this having been recognized long ago by Seneca through his famous “*errare humanum est.*” What is newer is the fact that errors are largely dealt with at different levels of information processing: prevention, detection, inhibition, correction and, if these mechanisms finally failed, strategic behavioral adjustments after errors.

The intrinsic proneness of humans to commit errors indicates that, after failures due to human errors (in industry, transportation …), adding new prescriptions is probably of little use. A more efficient attitude could be to ask oneself whether or not some situations, tasks or material, due to the nature or human information processing system, would favour human errors. Moreover, it could also be wondered whether some slight modifications of a task would allow operators to correct their errors, since the human brain is equipped of a rather efficient action monitoring system which might allow certain failures to be avoided.

Teachers, may be, should not be that severe as regard errors. Errors sometimes reflect low involvement, negligence or low motivation but, often, they simply reflect the nature of the human information processing system. It seems that errors should, on the contrary, be used for teaching efficient correction and how to avoid them in the future. Finally, an error by itself is often assumed to be a very efficient teacher because learning from past mistakes is essential to ensure future successful behavior (e.g., Holroyd and Coles, 2002). It has been proposed that the Ne reflects a key mechanism for this type of learning from errors (Holroyd and Coles, 2002).

Now, it could be hoped that, in specific situations, if precise enough instructions or long enough teaching is provided, errors could be completely eradicated. Although, from what precedes, one can cast some doubts regarding the results, we wonder whether one should hope complete eradication of errors. Indeed, it is possible that certain general principles of organization are efficient whatever the considered organization level. If this were the case, one could wonder whether some principles of organization of the sensorimotor system could find correspondences in other domains where reliability is also critical. We will try a comparison with the apparently remote domain of DNA replication.

The high (but not perfect) reliability of DNA replication mechanisms is not only dependent of complementary base-pairing. It also needs different “proofreading” enzymatic mechanisms that sequentially correct mispairing, when present (Alberts et al., 2002).

Complementary base-pairing is not extremely reliable and may produce several initial mispairing. However, certain of these initial mispairing are rendered impossible by DNA polymerase itself which “double-checks” the base-pair configuration before this enzyme catalyzes the covalent binding of mispaired nucleotides. In a second step, if this double-checks mechanism has failed and an incorrectly paired base has been covalently bound to the growing strand, a separate subunit or a separate domain of DNA polymerase, acting as a “self-correcting” system (called “exonucleolytic proofreading”), will correct several of these incorrect pairings. Now, if this self-correcting mechanism also fails, another and last proofreading mechanism (called “strand-directed mismatch repair”) will detect most remaining anomalous pairings and correct them. The end product of DNA replication is, finally, highly reliable and results in a very small proportion of errors (Alberts et al., 2002).

In our terms one could say that the replication process initially engages a preventive (proactive) mechanism with DNA polymerase double-checks. If this proactive mechanism fails, two successive reactive proofreading ones will detect and correct most remaining incorrect base-pairings. [which we could qualify as partial (replication) errors, since covalent binding has already occurred]. Full blown errors are those, when present, which escaped prevention, detection, and correction by the enzymatic replication monitoring system. This enzymatic monitoring system is to be considered as a very sophisticated mechanism which has evolved so efficiently that it powerfully secures the replication process. One might also consider that this system is not completely perfect, yet (since some errors occasionally still occur) and hope that, after a few million more years of evolution, full replication errors will be completely eradicated. However, this should not be: it is clear that maintaining a certain level of errors constitutes a supreme refinement of evolution, without which any adaptation of living beings to changing environments would be impossible. Eradicating completely errors would mean the assured future death of all living species.

If we accept to consider that sensorimotor activities seem to obey similar rules as DNA duplication does, it could also well be that errors should not be eradicated either in sensorimotor activities. Errors might constitute a behavioral avatar of biological variability. This variability might allow exploring new behaviors. Of course, most of these new behaviors reveal to be inappropriate, but some can occasionally reveal to be perfectly fit to an unnoticed change of the environment. In this latter case, these new behaviors, discovered by error, may become the new norm and could even be taught to others. As a consequence, teachers should not be completely negative as regards errors because (1) errors probably contribute to shape efficient and long-living new behaviors, which constitute a sort of self random learning, and (2) errors, even when they do not reveal new appropriate behavior, trigger behavioral adaptations (such as, for example post-error slowing) aimed at avoiding the same kind of errors in the future. This implies that errors, when produced in a given context where they are not too costly, might help avoiding future errors in more costly contexts. As such, errors might be exploited in a teaching perspective. Therefore, teachers should, to a certain extent, recognize and try to exploit the virtues of errors.

## Conflict of Interest Statement

The Guest Associate Editor Mireille Besson declares that, despite being affiliated to the same institution as the authors, the review process was handled objectively and no conflict of interest exists. The authors declare that the research was conducted in the absence of any commercial or financial relationships that could be construed as a potential conflict of interest.

## Footnotes

1 Automatic capture and controlled orientation may also be referred to as “bottom up” and “top down” control of attention, respectively, in the literature (e.g., Buschman and Miller, 2007).

2 Although more complex features (such as closure), or even extensive practice may result in “pop out” effects (reviews in Treisman, 1996; Hochstein and Ahissar, 2002), which stress, in the latter case, the importance of learning.

3 We will justify this assumption in the “intrinsic factors” section.

4 Remembering the distinction between “involuntary” and “effortless” meanings of the term “automatic” (Regan, 1981), the important point here is that the congruent response is triggered involuntarily since “once learned, an automatic process is difficult to suppress, to modify, or to ignore” (Schneider and Shiffrin, 1977, p. 2).

5 In this case, it is likely that the side of the jump is chosen in advance while the hand (or foot) stopping movement is determined after.

6 Although effects of time preparation on central processes cannot be excluded, this idea is, to our opinion, less grounded yet; therefore we will not present or discuss it here.

7 This effect holds for foreperiods superior to 200 ms otherwise, there is no time enough to get prepared (Bertelson, 1967).

8 The Ne must not be confounded with the feedback-related negativity (FRN: Miltner et al., 1997) which may be elicited by error feed-backs.

9 The Ne begins about 30 ms after EMG onset but before the mechanical response.
